# Laparoscopic resection of retroperitoneal lymphangioma around the pancreas: a case report and review of the literature

**DOI:** 10.1186/s13256-015-0760-z

**Published:** 2015-12-10

**Authors:** Takafumi Sato, Yoichi Matsuo, Kazuyoshi Shiga, Kenta Saito, Mamoru Morimoto, Hirotaka Miyai, Hiromitsu Takeyama

**Affiliations:** Department of Gastroenterological Surgery, Nagoya City University Graduate School of Medical Sciences, 1, Kawasumi, Mizuho-cho, Mizuho-ku, Nagoya, 467-8601 Japan

**Keywords:** Cystic lymphangioma, Retroperitoneum, Laparoscopy

## Abstract

**Introduction:**

Lymphangiomas are rare, benign tumors. An intra-abdominal location of these lesions is rarer still and there are only a few reports describing laparoscopic resection for retroperitoneal lymphangiomas, especially in tumors that mimic pancreatic tumors.

**Case presentation:**

We present the case of an asymptomatic 30-year-old Japanese woman in whom a cystic tumor was found incidentally in close approximation to the pancreas. Because the tumor was located in the retroperitoneal space and the body of the pancreas was compressed, we were unable to distinguish a cystic lymphangioma from cystic pancreatic tumors. We started the procedure laparoscopically with five ports. The tumor was in fact separated from the pancreas and was dissected free from the body of the pancreas using scissors and laparoscopic coagulating shears. The left gastric vessels, which were compressed by the tumor, were preserved. As we realized that the tumor was connected to the retroperitoneal lymphatic tissue, we completed the procedure by performing a cystectomy without rupture. The specimen was extracted using a plastic bag. Our patient was discharged on postoperative day 7 without any complications. There is no evidence of recurrence during a >2-year observation period.

**Conclusions:**

In addition to the therapeutic significance in differentiating between a cystic lymphangioma in close approximation to the body of the pancreas and a pancreatic cystic neoplasm, the laparoscopic approach is feasible and effective.

**Electronic supplementary material:**

The online version of this article (doi:10.1186/s13256-015-0760-z) contains supplementary material, which is available to authorized users.

## Introduction

Lymphangiomas are rare and benign tumors, which are caused by a malformation or a blockage of lymphatic vessels [1]. While in most cases the lesions are located superficially on the body, on rare occasions they can occur in the retroperitoneal space [2, 3]. There are two types of lymphangiomas, namely cystic and cavernous. If the tumor is located adjacent to the pancreas, it is very difficult to diagnose through preoperative imaging alone [4]. Because recurrence after drainage of a cystic lymphangioma has been reported [5], complete surgical resection is preferred for a cure [1]. However, some authors argue that an adequate resection for lymphangioma is a cystectomy and that extended resection should be avoided [1, 2]. Here we describe the case of a patient with cystic lymphangioma, which arose from retroperitoneal lymphatic vessels around the superior edge of the pancreas.

## Case presentation

**Fig. 1:**
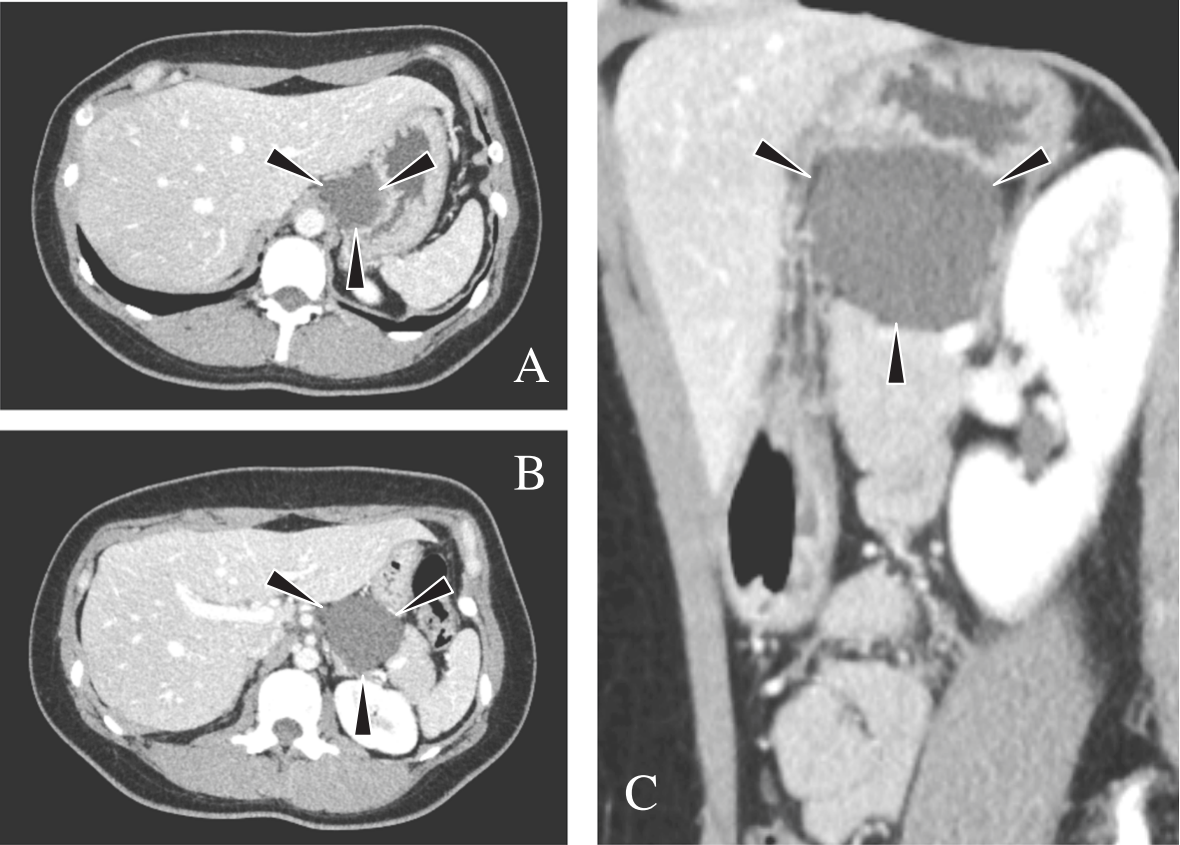
Preoperative contrast-enhanced computed tomography images. **a** and **b** are axial images. **c** is a sagittal image. The *arrows* show the cystic tumor. The wall is not enhanced and the tumor is abutting the lesser curvature of the stomach (**a** and **c**), and the body of the pancreas (**b** and **c**)

A 30-year-old Japanese woman presented to our hospital with chronic cough and had a workup in the internal medicine department. She had a past history of hyperlipidemia. A chest computed tomography scan showed a cystic lesion attached to the body of her pancreas, and a diagnosis of cystic pancreatic tumor was made. Blood tests showed neither inflammation nor anemia. Her serum amylase, lipase, aminotransferase, and bilirubin levels were within normal limits. Tumor markers including CEA, CA19-9, and AFP were not elevated. A contrast-enhanced computed tomography scan of her abdomen showed a 5 cm tumor (Fig. 1). The lesion was attached to and compressing the body of the pancreas, the left gastric artery, the body of the stomach, and the abdominal part of the esophagus superiorly. The wall of the mass was thin and not enhanced. Magnetic resonance images showed a multilocular tumor. Neither an internal nodule nor septations were noted. There was no abnormality in the pancreatic duct, and the main pancreatic duct was not in communication with the lesion. Endoscopic ultrasonography showed that the cyst content had iso- and hypoechoic density with a thin wall and no mural nodules. A fine-needle aspiration biopsy was not performed. Differential diagnosis included a mucinous cystic neoplasm, a pancreatic cyst, or a retroperitoneal cystic lymphangioma. She was thus referred to the surgical department for evaluation. At this stage, we could not determine whether a pancreatectomy was necessary, thus we proceeded with a diagnostic laparoscopy.

The surgery was performed 1 month after the first visit to our hospital. Under general anesthesia, the operation was started laparoscopically with five ports (5 mm × 3 and 12 mm × 2). The tumor was within the lesser sac, which was incised and a window created. Because the left lateral segment of the liver was large and obscured the surgical view, we added a 5-mm port to elevate the lateral segment. The tumor was located in the retroperitoneal space and was attached to the body of the pancreas. We began the dissection of the tumor from the opposite side of the pancreas using laparoscopic coagulation shears (LCS, Harmonic ACE^®^; Ethicon Endo-Surgery, Cincinnati, OH, USA) and scissors. Branch vessels from the left gastric artery and vein were divided and the compressed left gastric vessels were preserved. The dissection was done easily and bluntly. We dissected the tumor from the pancreas gently using a lymph node dissection technique similar to that in gastric cancer, and were able to separate the mass from the pancreas. Eventually, the tumor was connected only to retroperitoneal lymphatic tissue around the celiac axis. At this stage it became clear that this was a case of cystic lymphangioma; as such, we finished the operation with cystectomy only. The specimen was extracted using a plastic bag (Endo-pouch^®^; Ethicon Endo-Surgery). An additional movie file shows this image in more detail (see Additional file 1). The postoperative course was uneventful and our patient was discharged on the sixth postoperative day. As the postoperative follow-up, our patient received an abdominal computed tomography examination at 3 months after surgery and then every 6 months. Our patient had no signs of recurrence for a 2-year period after the surgery.

**Fig. 2:**
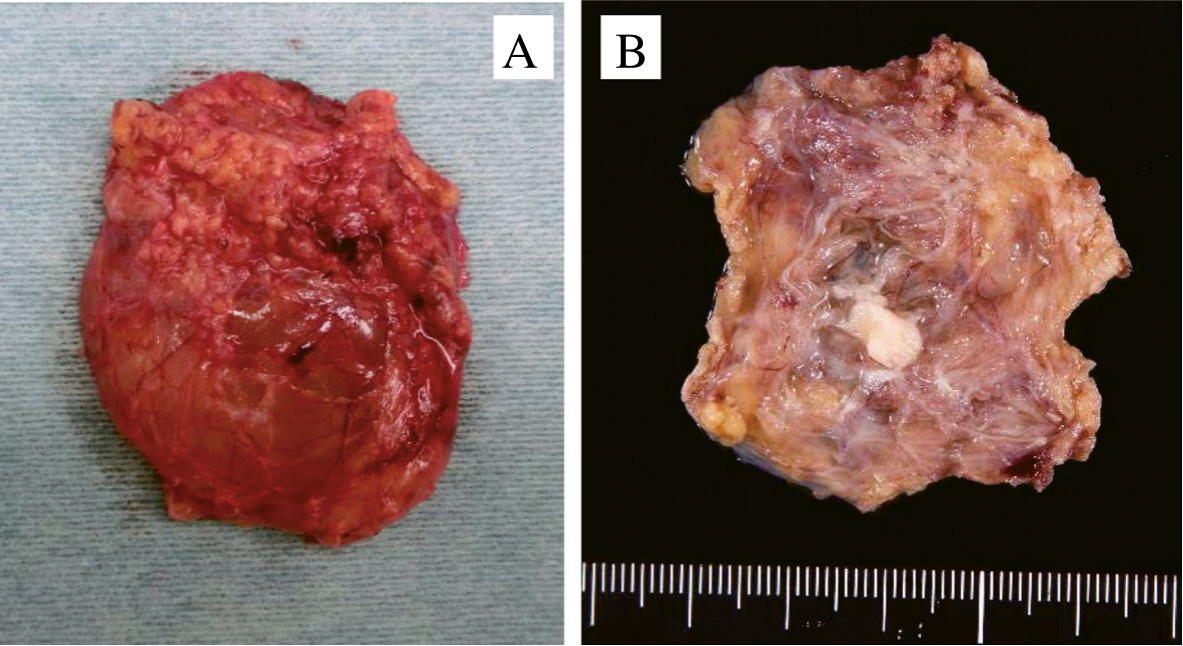
**a** Resected specimen, gross view. Cyst fluid was serous and clear. **b** Internal aspect of the formalin-fixed specimen

**Fig. 3:**
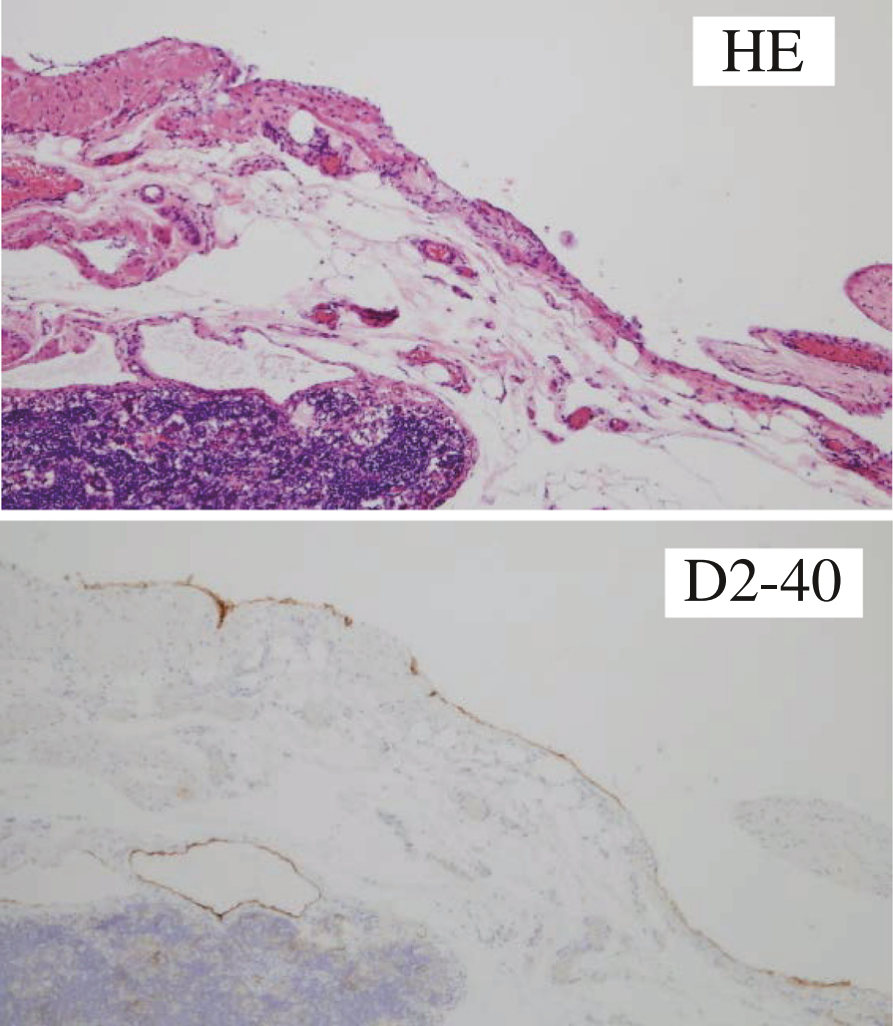
Hematoxylin and eosin staining and immunostaining for D2-40. Internal aspect of the wall is lined with D2-40-positive endothelial cells

A histopathological examination showed that the cyst was lined by flattened endothelium. Immunostaining was positive for CD31, D2-40, AE1/AE3, and SMA. Other markers including CD34 and calretinin were negative. The diagnosis was made of a retroperitoneal cystic lymphangioma (Figs. 2 and 3).

## Discussion

A cystic tumor in the retroperitoneal space has many potential diagnoses, particularly when located adjacent to the pancreas, in which case the decision to operate is more difficult [4]. Lymphangiomas generally require only cystectomy; pancreatic tumors, on the other hand, usually require pancreatectomy, which is a far more involved operation.

Lymphangiomas are benign lesions characterized by proliferating lymphatic vessels, and are classified as either hamartomas or lymphangiectasis. Since lymphangiomas are tumors that develop before birth, in most cases they are discovered when patients are young [6]. Adult cases are rare and retroperitoneal lesions are rarer still [4]. In the present case, the tumor was located in the retroperitoneal space and abutting the pancreas; we were therefore unable to make a preoperative differential diagnosis between pancreatic tumors, such as a pancreatic pseudocyst or a mucinous cystic neoplasm, and a lymphangioma based on radiology alone.

Makni *et al*. espouse the concept that an extended resection should not be performed for benign lymphangiomas [2]. In the last 10 years (since 2005), 31 cases of retroperitoneal lymphangioma were reported. Cystectomy was performed in 21 cases, pancreaticoduodenectomy in one case, distal pancreatectomy with splenectomy in four cases, adrenalectomy in two cases, and miscellaneous abdominal operation in one case (two reports did not describe details of the surgical procedure). If surgery is undertaken for a retroperitoneal mass of unknown cell origin, the surgeon must be able to modify the extent of surgery based on the nature of the tumor as observed intraoperatively in an attempt to preserve the integrity of any adjacent organs. To that end, a laparoscopic approach is useful for diagnosing and sometimes treating such lesions.

The first report of laparoscopic resection for retroperitoneal lymphangioma was published by Targaroma *et al*. in 1994 [7]. Subsequently, another case was reported in the setting of the advancement of laparoscopic techniques and instruments. To date, there are only eight reports describing ten cases with laparoscopic resection and two reports describing retroperitoneoscopic resection for retroperitoneal lymphangiomas in adult patients [2, 7–15]. Table 1 lists all of the reported cases, including our report. The median age was 45 years old (range; 25–79) and median size of the tumor was 10 cm (range; 3–18). The male to female ratio was 2:11. These reports showed the usefulness of a laparoscopic approach for retroperitoneal lymphangiomas; this approach can also be useful for diagnosis. For example, when the tumor is located on the superior border of the pancreas in the retroperitoneal space, as in our case, we can resect the tumor using the same technique used for lymph node dissection for gastric cancer without damaging the tumor and pancreas. In patients with lymphangiomas, more precise techniques than those of lymph node dissection are required because the wall of the lymphangioma is weak and fragile. For this purpose, the magnification inherent in laparoscopy makes for easier identification.

**Table 1:** Previous reports on laparoscopic/retroperitoneoscopic resection for retroperitoneal cystic lymphangiomas

Author	Year	Age	Gender	Symptom	Size (cm)	Approach
Tagarona *et al*. [7]	1994	45	F	Abdominal pain and palpable mass	12	Laparoscopic
Tsukamoto *et al*. [14]	2003	36	F	Hypochondralgia and back pain	11	Laparoscopic
Celia *et al*. [12]	2007	25	F	Abdominal pain	7	Laparoscopic
Trindade *et al*. [13]	2007	68	M	Abdominal pain	6	Laparoscopic
Kasza *et al*. [15]	2010	52	M	Back pain	10	Laparoscopic
Yagihashi *et al*. [10]	2011	38	F	Hypochondralgia	18	Retroperitoneoscopic
Makni *et al.* [2]	2012	37	F	Abdominal pain	11	Laparoscopic
		48	F	None (incidental finding)	5	Laparoscopic
		30	F	Abdominal pain	10	Laparoscopic
Black *et al*. [9]	2013	66	F	Abdominal pain	5	Laparoscopic
Liu *et al.* [11]	2013	45	F	None (incidental finding)	3	Retroperitoneoscopic
Jung *et al*. [8]	2014	79	F	Epigastric discomfort, poor oral intake	13	Laparoscopic
Present case	2015	30	F	None (incidental finding)	5	Laparoscopic

## Conclusions

A retroperitoneal lymphangioma in close approximation to the pancreas is easily and safely resected laparoscopically. In addition to the therapeutic significance in differentiating between a cystic lymphangioma in close approximation to the body of the pancreas and a pancreatic cystic neoplasm, the laparoscopic approach is feasible and effective.

## Consent

Written informed consent was obtained from the patient for publication of this case report and any accompanying images. A copy of the written consent is available for review by the Editor-in-Chief of this journal.

## Additional file

Additional file 1: **Laparoscopic resection of retroperitoneal lymphangioma around the pancreas.** Movie file of our procedure. (M4V 20211 kb)
